# Hyponatremia is not induced by postoperative hypotonic fluids in infants with biliary atresia after sufficient diuresis

**DOI:** 10.1111/ped.70016

**Published:** 2025-05-14

**Authors:** Kazuki Yokota, Hiroo Uchida, Chiyoe Shirota, Takahisa Tainaka, Wataru Sumida, Satoshi Makita, Hizuru Amano, Yoichi Nakagawa, Takuya Maeda, Yousuke Gohda, Daiki Kato, Akinari Hinoki

**Affiliations:** ^1^ Department of Pediatric Surgery Nagoya University Graduate School of Medicine Nagoya Aichi Japan; ^2^ Department of Pediatric Surgery Aichi Developmental Disability Center Central Hospital Kasugai Aichi Japan

**Keywords:** biliary atresia, diuresis, fluid therapy, hyponatremia, infant

## Abstract

**Background:**

In Japan, the administration of extra‐hypotonic fluids (approximately 35 mmol/L of sodium) as maintenance fluid is still the mainstream practice, and there have been relatively few reports on maintenance intravenous fluid therapy. Since 2014, our institution has administered maintenance fluids containing 83 mmol/L of Na (HALF) after diuresis is achieved post‐Kasai portoenterostomy for biliary atresia (BA). We investigated whether hyponatremia is induced by the administration of half saline during postoperative maintenance of infants with BA.

**Methods:**

Patients who underwent surgery for BA at our institution were included. The serum sodium concentration ([Na]) before and after surgery and the incidence of hyponatremia were compared between patients administered fluids with [Na] of 35 mmol/L (exHYPO group, 59 patients) and those with [Na] of 83 mmol/L (HALF group, 20 patients).

**Results:**

The median age of patients was 59 days. There were no significant differences in the background or preoperative [Na] between groups. There was a significant decrease in [Na] on postoperative day 3 (POD3) in the exHYPO group compared with the preoperative [Na] value in the exHYPO group and the [Na] value on POD3 in the HALF group. There were no significant differences in [Na] before and after surgery in the HALF group. The odds ratio was 21.0, and the 95% confidence interval was 3.31–130, indicating that the exHYPO group had an increased risk of hyponatremia.

**Conclusion:**

Administration of half saline as maintenance fluid can maintain [Na] levels during postoperative care of infants with BA.

## INTRODUCTION

In 1957, Holliday and Segar reported a standard fluid management strategy for pediatric patients, which has since been adopted worldwide for a prolonged period.^1^ They recommended that the water requirement for children is 100 mL/kg/day for the first 10 kg, plus 50 mL/kg/day for the next 10 kg, plus 20 mL/kg/day for each remaining kg and that the maintenance requirements of sodium (Na) and potassium (K) are 3.0 and 2.0 mEq/100 mL/day, respectively. Thus, an extra‐hypotonic fluid of approximately 0.2% saline has been used as maintenance intravenous fluid.

Recently, the use of extra‐hypotonic fluids has come under scrutiny. Several reports have stated that the administration of extra‐hypotonic fluids could lead to hyponatremia^2^, ^3^, ^4^ because hospitalized children have a non‐osmotic stimulus for antidiuretic hormone (ADH) secretion. The non‐osmotic stimulus of ADH secretion is induced by positive pressure ventilation, stress, nausea, vomiting, hypoglycemia, fever, and decreases in intravascular volume, all possible associations or sequelae of illness or surgery.^5^ Au et al. previously reported that children undergoing surgical procedures are at risk of developing hyponatremia, which could lead to unrecognized hypovolemia, free‐water administration, sodium loss, cerebral salt wasting, secretion of ADH, redistribution of extracellular water, and changes in cellular homeostasis.^6^ In the last decade, many randomized, controlled studies and meta‐analyses of hypotonic versus isotonic fluids in hospitalized children with or without surgery have been reported,^5^, ^7^, ^8^, ^9^, ^10^, ^11^, ^12^, ^13^, ^14^, ^15^ with results showing that the administration of extra‐hypotonic fluids tended to cause hyponatremia. Isotonic fluids are recommended as maintenance fluid for postoperative pediatric patients to prevent iatrogenic hyponatremia, even after sufficient diuresis is achieved following the postoperative invasive period. However, there is no report on maintenance fluid focusing only on infants postoperatively.

In Japan, the administration of extra‐hypotonic fluids (approximately 35 mmol/L of Na) as maintenance fluid is still the mainstream practice, and there have been relatively few reports concerning maintenance intravenous fluid therapy.^16^ At our institution, until 2013, maintenance fluids containing 35 mmol/L of Na (exHYPO) were administered after diuresis was achieved post‐Kasai portoenterostomy for biliary atresia (BA); however, since 2014, fluids containing 83 mmol/L of Na (HALF) are being administered. Because BA is highly liver‐damaging, especially to postoperative liver function, we sought to investigate whether serum sodium in half saline would be acceptable, considering that physiological saline increases ascites.

In this study, we retrospectively investigated patients with BA to determine whether half saline induces hyponatremia as a maintenance fluid, even after sufficient diuresis is achieved.

## METHODS

We retrospectively reviewed the medical records of patients who underwent Kasai portoenterostomy for BA between January 2003 and December 2017. During this period, we performed the Kasai procedure on 94 patients with BA in our institution. We defined hyponatremia as a sodium concentration ([Na]) less than 136 mmol/L, which represents the lower limit of the normal range at our institution. There were 79 patients with a preoperative serum [Na] of ≥136 mmol/L, and these 79 patients were included in this study. The remaining 15 patients, who had hyponatremia with [Na] less than 136 mmol/L preoperatively, were excluded.

Our standard postoperative intravenous maintenance fluid management strategy is as follows: Isotonic fluid is first administered after surgery is completed. This is changed to maintenance fluid approximately 12 h later, when diuresis develops, indicating that the postoperative invasive period has passed and residual intraoperative bodily fluid deficits have been compensated for. For patient management after surgery for BA, the basic infusion volume is set at 150 mL/kg/day. On and after postoperative day (POD) 3, oral intake is started, and the infusion volume is decreased gradually. Steroids are not administered during this period.

The maintenance fluid used at our institute was exHYPO until 2013; however, since 2014, HALF has been administered. exHYPO comprises 35 mmol/L of Na, 20 mmol/L of K, and 35 mmol/L of chlorine (Cl) with 7.5% glucose solution, whereas HALF comprises 83 mmol/L of Na, 12 mmol/L of K, and 109 mmol/L of Cl with 3.75% glucose solution (Table 1).

**TABLE 1:** Composition of the maintenance fluid.

	exHYPO^a^	HALF^b^
Na (mmol/L)^c^	35	83
K (mmol/L)^d^	20	12
Cl (mmol/L)^e^	35	109
Glu (%)^f^	7.5	3.75

^a^ exHYPO: maintenance fluid with a sodium concentration of 35 mmol/L.

^b^ HALF: maintenance fluid with a sodium concentration of 83 mmol/L.

^c^ Na: sodium.

^d^ K: potassium.

^e^ Cl: chlorine.

^f^ Glu: glucose.

The following information was recorded from patients' medical records: sex, age at surgery, types of maintenance fluid (exHYPO or HALF), administration of postoperative diuretics or blood transfusions, and [Na] preopertaively and on the first and third POD.

In this study, we compared the exHYPO and HALF groups with respect to the frequency of hyponatremia and changes in [Na] pre‐ and postoperatively.

Univariate analyses were performed using Fisher's exact test for categorical variables, and the Mann–Whitney *U*‐test was used for continuous variables. *p*‐values <0.05 were considered statistically significant. This retrospective survey was approved by the ethics committee of the Nagoya University Hospital (approval number: 2017–0196).

## RESULTS

Seventy‐nine patients with normal preoperative [Na] who underwent primary Kasai portoenterostomy for BA were included in this study. Table 2 shows the patient characteristics. There were 59 patients in the exHYPO group and 20 in the HALF group. In the exHYPO group, there were 26 males (44%), and the median age and weight at surgery were 61 (range, 28–129) days and 4.66 (range, 2.16–6.82) kg, respectively. The median [Na], blood glucose levels and serum creatinine levels before surgery were 138 (range, 136–142) mmol/L, 88 (55–176) mg/dL, and 0.18 (0.10–0.32) mg/dL, respectively. In the HALF group, there were six males (30%), and the median age and weight at surgery were 57 (range, 29–105) days and 4.52 (range, 2.67–6.19) kg, respectively. The median [Na], blood glucose levels and serum creatinine levels preoperatively were 138 (range, 136–142) mmol/L, 85 (49–178) mg/dL and 0.18 (0.09–0.32) mg/dL, respectively. There were no significant differences in these four factors between both groups. Transfusions including red blood cells and fresh frozen plasma were performed in four patients in the HALF group and three patients in the exHYPO group, and no significant difference was observed between both groups (*p* = 0.064). Postoperative diuretics were administered to five patients in the HALF group and two patients in the exHYPO group, with a significant difference between both groups (*p* = 0.010). The infusion volume of maintenance fluid up to the third POD was 150 mL/kg/day in all cases.

**TABLE 2:** Patient characteristics and background.

	exHYPO^c^	HALF^d^	*p*‐Value
Total, *n*	59	20	
Males, *n* (%)	26 (44)	6 (30)	0.30^b^
Age at surgery (days), median (range)	61 (28, 129)	57 (29, 105)	0.33^a^
Weight at surgery (kg), median (range)	4.66 (2.16, 6.82)	4.52 (2.67, 6.19)	0.33^a^
Preoperative [Na] (mmol/L), median (range)^e^	138 (136, 142)	138 (136, 142)	0.08^a^
Preoperative blood glucose levels (mg/dL), median (range)	88 (55, 176)	89.5 (63, 160)	0.74^a^
Preoperative serum creatinine levels (mg‐dL), median (range)	0.18 (0.10, 0.32)	0.20 (0.10, 0.29)	0.22^a^
Postoperative blood transfusion, *n* (%)^f^	3 (5.1)	4 (20)	0.064^b^
Postoperative diuretic administration, *n* (%)	2 (3.4)	5 (25)	0.010^b^

^a^ Mann–Whitney *U*‐test.

^b^ Fisher’s exact test.

^c^ exHYPO: maintenance fluid with a sodium concentration of 35 mmol/L.

^d^ HALF: maintenance fluid with a sodium concentration of 83 mmol/L.

^e^ [Na]: serum sodium concentration.

^f^ Transfusion includes red blood cells and fresh frozen plasma.

**FIGURE 1:**
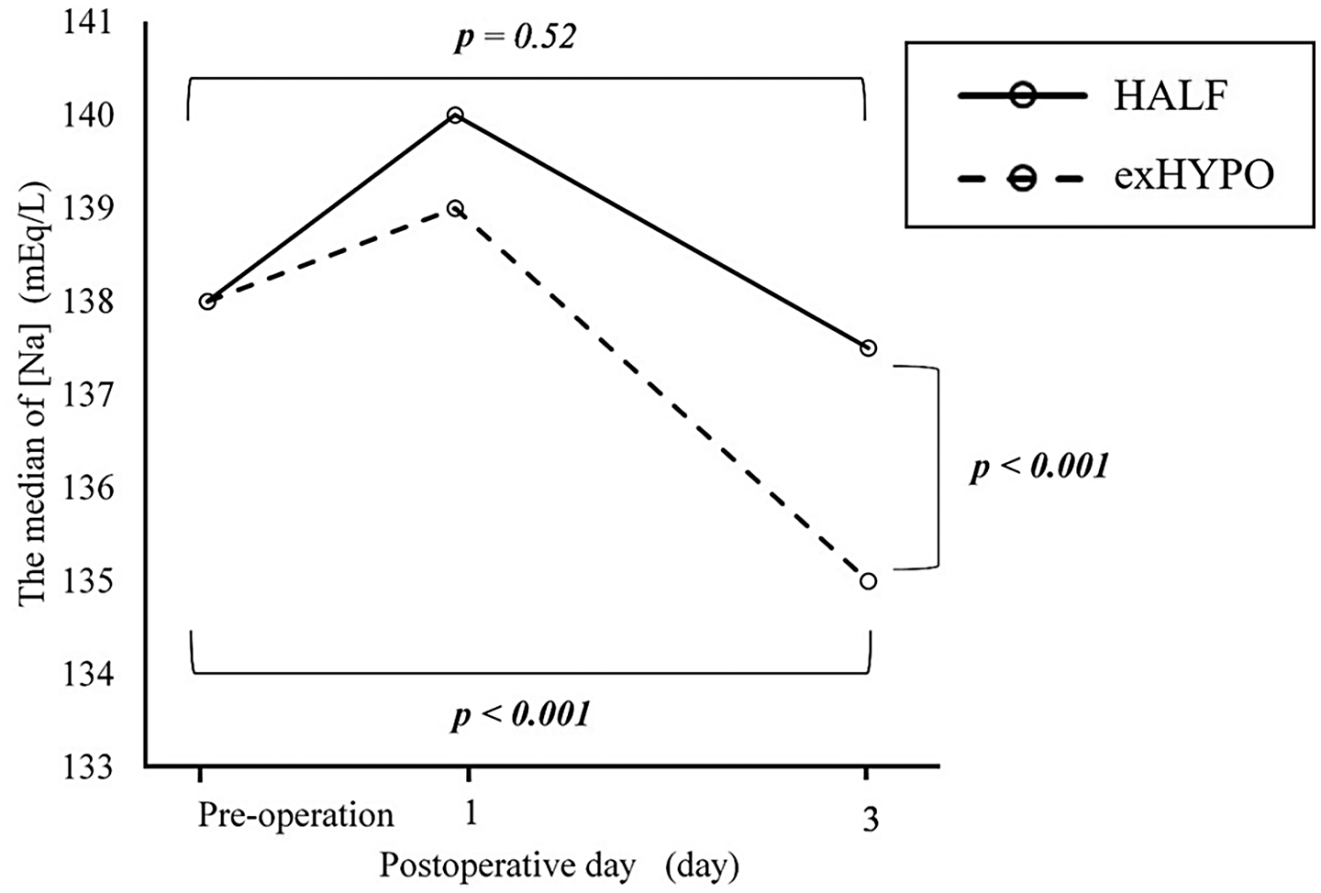
Changes in serum sodium concentration [Na] in the HYPO group (maintenance fluid with a sodium concentration of 35 mmol/L) and HALF group (maintenance fluid with a sodium concentration of 83 mmol/L).

Figure 1 shows the postoperative [Na] and change in [Na] pre‐ and postoperatively. In the exHYPO and HALF groups, the median [Na] at POD1 were 139 (range, 135–144) mmol/L and 140 (range, 137–145) mmol/L, respectively. At POD3, the values were 135 (range, 132–149) mmol/L and 137.5 (range, 135–141) mmol/L, respectively. There were significant differences between both groups (*p* < 0.001). Changes in the absolute value of [Na] were −2 mmol/L in the exHYPO group and −1 mmol/L in the HALF group, representing a significant difference (*p* = 0.005). On POD3, [Na] in the exHYPO group was significantly decreased compared with preoperative [Na] (*p* < 0.001), whereas in the HALF group, there were no significant differences in [Na] between the preoperative levels and those on POD3 (Figure 1). Changes in [Na] and significant differences between the exHYPO and HALF groups are shown graphically in Figure 1.

Table 3 presents various data for fluid management. The blood glucose levels were significantly higher in the exHYPO group. There were no significant differences in osmolality before and after surgery; however, a significant difference was observed in the body weight ratio (*p* = 0.003). No patients developed renal dysfunction either before or after surgery.

**TABLE 3:** Data on various values on postoperative day 3.

	exHYPO^b^	HALF^c^	*p*‐Value
Blood glucose levels (mg/dL), median (range)	97 (52, 168)	85 (49, 178)	0.00005^a^
Weight (kg), median (range)	4.34 (2.20, 6.69)	4.47 (3.55, 5.63)	0.9^a^
Pre‐ and postoperative weight ratio	0.95 (0.89, 1.01)	0.99 (0.86, 1.03)	0.0003^a^
Pre‐ and postoperative osmotic pressure ratio	0.99 (0.96, 1.02)	0.99 (0.94, 1.03)	0.309^a^
Serum creatinine levels (mg‐dL), median (range)	0.17 (0.1, 0.31 9)	0.18 (0.09, 0.32)	0.29^a^

^a^ Mann–Whitney *U*‐test.

^b^ exHYPO: maintenance fluid with a sodium concentration of 35 mmol/L.

^c^ HALF: maintenance fluid with a sodium concentration of 83 mmol/L.

We further investigated the frequency of hyponatremia. On POD3, 31 of 59 (53%) patients in the exHYPO group and 1 of 20 (5%) in the HALF group had hyponatremia. Comparing these two groups, the odds ratio was 21.0 and the 95% confidence interval was 3.31–130, indicating that the exHYPO group was at an increased risk of hyponatremia (Table 4).

**TABLE 4:** Frequency of hyponatremia on postoperative day 3.

	[Na] ≥136 mmol/L, *n* ^a^	[Na] <136 mmol/L, *n*	*p*‐Value	OR^b^ (95% CI^c^)
exHYPO^d^	28	31	*p* < 0.001	21.0 (3.31–130)
HALF^e^	19	1		

^a^ [Na]: serum sodium concentration.

^b^ OR: odds ratio.

^c^ CI: confidence interval.

^d^ exHYPO: maintenance fluid with a sodium concentration of 35 mmol/L.

^e^ HALF: maintenance fluid with a sodium concentration of 83 mmol/L.

We examined only cases of laparoscopic surgery to consider the impact of invasiveness. Laparoscopic surgery was performed in 28 cases, with 10 cases in the exHYPO group and 18 cases in the HALF group. There was no significant difference in preoperative [Na] between the two groups. However, on the POD3, the median [Na] was 135.5 (range: 133–139) mmol/L in the exHYPO group and 137.5 (range: 136–141) mmol/L in the HALF group (*p* = 0.005), showing a significant difference. Additionally, the incidence of hyponatremia on POD3 was 5 of 10 (50%) patients in the exHYPO group and 1 of 18 (5.6%) patient in the HALF group (*p* = 0.006), also showing a significant difference.

The postoperative reduction rate of serum bilirubin level and 1‐year native liver survival rate were 72% and 69% in the exHYPO group, and 75% and 75% in the HALF group, with no significant differences between the two groups.

## DISCUSSION

In the present study, we showed that for Japanese infants with BA, hyponatremia could be prevented when half saline was administered as a maintenance fluid after the postoperative invasive period. Hyponatremia has been reported to occur frequently, even with the administration of half saline, and it was suggested that the administered maintenance fluid should be isotonic.^5^, ^10^, ^13^, ^14^, ^17^ However, in our study, there was only one case of hyponatremia in the HALF group, and half saline was effective in preventing hyponatremia. The reason for this may be that the addition of isotonic fluid during the acute phase of the postoperative period (12 h postoperatively) compensates for the loss of sodium, whereas previous reports have administered hypotonic electrolyte maintenance fluids immediately postoperatively. In the present study, [Na] increased on POD1 in both groups, as both groups received the initial administration of isotonic fluid. Approximately 12 h later, when diuresis develops and residual intraoperative bodily fluid deficits have been compensated for, we switch to maintenance fluid. In the exHYPO group, a significant decrease in [Na] was observed on POD3 compared with the preoperative [Na], whereas the HALF group showed no significant decrease in [Na]. Thus, the HALF group maintained homeostasis. The subsequent maintenance infusion could not maintain [Na] with exHYPO but could do so with HALF. In addition, regarding other data related to fluid management, the exHYPO group showed higher blood glucose levels, which is thought to be due to the concentration of glucose in the maintenance fluids (Table 1). Although there were no significant differences in the osmolality ratio before and after surgery between the two groups, the body weight ratio was significantly lower in the exHYPO group. This suggests that the circulating plasma volume in the exHYPO group may have been slightly insufficient. Infusion of isotonic fluids in patients with impaired liver function and hypoalbuminemia, as commonly observed in BA, is likely to induce ascites and edema. Therefore, the maintenance of [Na] with half saline would be highly beneficial for these patients. It would seem that a more hypotonic infusion than half saline would not preserve [Na]. Our study showed that postoperative hyponatremia was more frequently induced by the administration of hypotonic fluids than by that of half saline. The odds ratio was extremely high at 21.

Generally, transfusions and administration of diuretics affect [Na]. We found no significant difference in the proportion of patients who underwent transfusion in both groups, but a significant difference was observed in the proportion of patients who were administered diuretics. All diuretics administered were loop diuretics that do not usually reduce or affect [Na]. In this study, diuretics were frequently administered to the HALF group, but the HALF group still had higher [Na]. Accordingly, it was concluded that it is not necessary to consider the effects of diuretics.

ADH is considered to be involved in the etiology of hyponatremia when hypotonic fluid is administered. ADH release is regulated by osmotic stimuli and is also controlled by various non‐osmotic stimuli, including decreased extracellular fluid volume, pain, nausea, stress, surgery, and other factors. These non‐osmotic stimuli override osmotic control; therefore, the perioperative period is characterized by a high concentration of ADH, and the administration of hypotonic fluids will lead to hyponatremia.^5^, ^6^, ^18^ If hyponatremia is caused by the inappropriate secretion of ADH (SIADH), fluid overloading should increase the risk of hyponatremia; indeed, there are reports that hyponatremia is induced by an overdose of maintenance fluid.^8^, ^10^ Reducing the volume of administered hypotonic fluid prevents hyponatremia.^8^, ^10^ Our postoperative protocol, which comprises a relatively high volume of administration, such as 150 mL/kg/day, would easily induce hyponatremia. Moreover, as liver dysfunction is associated with hyponatremia,^19^ patients with BA may be prone to hyponatremia.

To our knowledge, there have been no prior reports focusing on postoperative maintenance fluid management in infants with BA. This is, thus, the first report demonstrating the efficacy of half saline fluid administration and risk of hypotonic fluid administration in young infants with BA. In our study, we focused on patients who underwent surgery for BA at a median age of 59 days. BA is a disease that develops only early in infancy; there were no significant differences in background factors, such as age, weight, and level of preoperative serum electrolytes in both groups. As the patients' background characteristics were homogeneous, we consider our findings useful for comparison.

The United Kingdom National Patient Safety Agency recommended the removal of 0.18% saline from hospital stock in 2007 to reduce the risk of iatrogenic hyponatremia.^2^ Additionally, the American Academy of Pediatrics published a guideline in 2018 recommending isotonic solutions as intravenous maintenance fluid therapy in children aged from 28 days to 18 years old.^20^ The National Institute for Health and Care Excellence issued an updated guideline in 2020 that suggested the use of isotonic fluid in term neonates over 8 days old.^21^ Although the guidelines recommend the use of isotonic fluid as intravenous maintenance fluid therapy rather than half saline, our study shows that [Na] can be maintained using HALF if the initial infusions are isotonic fluids, and our results differ from these recommendations.

A limitation of this retrospective study is that we did not measure the levels of regulatory hormones or osmotic pressure or perform urinalysis. If we had collected this information, it may have been possible to examine the association of hyponatremia with ADH level and other factors in more detail. To verify the proper maintenance fluid, we have initiated a randomized, controlled trial assessing isotonic fluid.

## CONCLUSIONS

The results of the present study show that, even if the postoperative invasive period has passed, the administration of extra‐hypotonic fluid as maintenance fluid cannot maintain [Na] in Japanese infants with BA. Therefore, extra‐hypotonic fluid administration is considered inappropriate as a maintenance strategy. In contrast, we found that if isotonic solution is administered well in the acute phase, [Na] can be maintained even if half saline is administered thereafter. Although isotonic solution is generally recommended for infusion, we observed no issues with HALF in our study. Therefore, it is beneficial to administer isotonic fluids in the acute postoperative period and half saline thereafter, particularly in the postoperative management of patients with impaired liver function, as in BA.

## AUTHOR CONTRIBUTIONS

K.Y., H.U., and A.H. designed the study protocol. K.Y., H.U., T.T., C.S., W.S., S.M., Y.N., T.M., Y.G., D.K., and A.H. obtained written, informed patient consent and collected data. K.Y., H.U., and H.A. analyzed the data. H.U., H.A., and A.H. conducted and supervised the study. K.Y., H.U., and H.A. wrote the first draft of the manuscript. All authors reviewed the manuscript. All authors read and approved the final manuscript.

## FUNDING INFORMATION

This research did not receive any specific grant from funding agencies in the public, commercial, or not‐for‐profit sectors.

## CONFLICT OF INTEREST STATEMENT

The authors declare no conflict of interest.

## ETHICS STATEMENT

This study was approved by the ethics committee of Nagoya University Hospital (approval number: 2017–0196; date of approval: September 6, 2017).

## INFORMED CONSENT

Informed consent was obtained from all participants by opt‐out method.

## CONSENT FOR PUBLICATION

Not applicable.

## Data Availability

The datasets used and/or analyzed during the current study are available from the corresponding author upon reasonable request.
